# SLC7A1 Overexpression Is Involved in Energy Metabolism Reprogramming to Induce Tumor Progression in Epithelial Ovarian Cancer and Is Associated with Immune-Infiltrating Cells

**DOI:** 10.1155/2022/5864826

**Published:** 2022-09-12

**Authors:** Shijing You, Xiaoying Zhu, Yao Yang, XiuZhen Du, Kejuan Song, Qingmei Zheng, Pengjiao Zeng, Qin Yao

**Affiliations:** ^1^Department of Obstetrics and Gynaecology, The Affiliated Hospital of Qingdao University, Qingdao, 266003 Shandong, China; ^2^Department of Gynecology, Qingdao Municipal Hospital, Qingdao, 266011 Shandong, China; ^3^Medical Research Center, The Affiliated Hospital of Qingdao University, Qingdao, 266000 Shandong, China

## Abstract

Cationic amino acid transporters (SLC7A1/CAT1) are highly expressed in human ovarian cancer (OC) tissues. However, the specific biological functions and mechanisms involved remain unclear. We used bioinformatics analysis to explore SLC7A1 expression level, prognostic value, and tumor mutation burden (TMB) in ovarian cancer (OC) tissues. We performed in vitro experiments to identify the expression and biological function of SLC7A1 in epithelial ovarian cancer (EOC) tissues and cells. An amino acid autoanalyzer was used to detect the effect of SLC7A1 on amino acid metabolism in EOC cells. Finally, SLC7A1 in OC was evaluated for cell-to-cell signalling and immune infiltration using online databases. We found that increased SLC7A1 expression in EOC cells and tissues was associated with poorer survival outcomes (*P* < 0.05) but not with tumor stage or grade of OC (*P* > 0.05). SLC7A1 is involved in the transport of phenylalanine and arginine in EOC cells, and its knockdown reduced the proliferation and migration of EOC cells and the resistance of cells to cisplatin. Furthermore, the TIMER database indicated that SLC7A1 overexpression was significantly positively correlated with levels of CD4^+^ memory resting cells, CD8^+^ effector memory cells, M0 macrophages, and cancer-associated fibroblasts (CAFs) in OC (*P* < 0.05) and significantly negatively correlated with CD4^+^ memory-activated cells (*P* < 0.05). Cell immunofluorescence indicated that SLC7A1 overexpression may affect the distribution of immune-infiltrating lymphocytes in tumors by inhibiting the expression of *CCL4*. Therefore, we concluded that SLC7A1 is involved in the metabolic remodelling of amino acids in EOC to promote tumor development and cisplatin resistance and is related to the tumor-infiltrating immune microenvironment of OC. SLC7A1 is a biomarker for predicting EOC progression and cisplatin resistance and represents a promising target for EOC treatment.

## 1. Introduction

As the most common malignant tumor type in gynecology, ovarian cancer (OC) is continuing to occur with greater frequency [1]. Due to the lack of specific clinical manifestations and reliable methods for early diagnosis, 60% to 70% of patients are diagnosed in the advanced stage (FIGO stage III or IV) [2]. Most patients with advanced OC have a 5-year survival rate of 29%, while patients with early OC have a survival rate of 92% [3]. Epithelial ovarian cancer (EOC) is the most common tumor type in ovarian cancer, accounting for 90% of new cases of ovarian cancer [4]. Currently, the standard treatment for epithelial ovarian cancer is surgery combined with platinum-based chemotherapy, but most patients experience relapse and metastasis due to chemotherapy resistance [5, 6]. Therefore, exploring the early diagnosis of EOC and revealing the key molecules that affect the sensitivity of platinum will help to improve the prognosis of EOC patients and improve the treatment effect of cisplatin.

Solute carrier family 7 (SLC7) is a group of membrane channel proteins that can be divided into the LAT and CAT families. L-type amino acid transporters (LATs) include SLC7A5-13 and SLC7A15, and cationic amino acid transporters (CATs) include SLC7A1-4 and SLC7A14. Members of the CAT family transport cationic amino acids primarily by facilitating diffusion of cellular substrates [7]. SLC7A2 is downregulated in ovarian cancer and can be used as a protective prognostic marker for OC [8]. The expression of SLC7A3 has been found to be significantly reduced in OC strains constructed to be cisplatin- and paclitaxel-resistant [9]. SLC7A4 and SLC7A14 have not been studied in OC. Only cationic amino acid transporter 1 (CAT1/SLC7A1) has been found to be upregulated in EOC, but it is unclear whether its high expression has a biological function.

SLC7A1 is expressed in most human tissues and mainly functions in transport of ornithine, lysine, and arginine [10, 11]. Current studies have shown that SLC7A1 is involved in tumor progression in liver, colorectal, and breast cancer [12–14]. In hepatoblastoma (HB), SLC7A1, as a substrate of the tumor suppressor gene *SPOP*, affects the progression of HB by regulating arginine metabolism, thus providing a new therapeutic target for HB [13]. Recent studies have shown SLC7A1 expression to be significantly higher in HGSOC than in normal ovarian tissue (*P* < 0.01) and negatively correlated with relapse-free survival (*P* < 0.05) [15]. Another study showed that SLC7A1-mediated arginine transport is involved in T cell proliferation and thus in the process of tumor immune escape [16]. However, it remains unclear whether SLC7A1 is involved in the amino acid metabolism of OC and the tumor immune microenvironment, and these questions are worthy of further exploration.

Therefore, our study addressed these problems and found that SLC7A1, a protein highly expressed in EOC, promotes tumor occurrence, development, and drug resistance. Knockdown of SLC7A1 affects amino acid metabolism in EOC. In addition, SLC7A1 may be involved in cancer immune lymphocyte infiltration through CCL4. Thus, the potential mechanism by which SLC7A1 operates in EOC was identified, which provides a new research direction for targeted therapy for treating EOC.

## 2. Materials and Methods

### 2.1. Cell Culture and Lentiviral Transfection

The EOC cell lines SKOV3 (RRID: CVCL_0532), OVCAR3 (RRID: CVCL_0465), and A2780 (RRID: CVCL_0134) and the normal human ovarian epithelial cell line IOSE80 (RRID: CVCL_5546) were obtained from the Institute of Biochemistry and Cell Biology, Chinese Academy of Sciences (Shanghai, China). All cell lines were identified by their source organization before purchase. IOSE80 cells were cultured in RPMI medium containing 15% fetal bovine serum (FBS, Gibco) and 1% penicillin/streptomycin, and EOC cells were cultured in DMEM containing 10% fetal bovine serum (FBS, Gibco) and 1% penicillin/streptomycin in a 5% CO_2_ incubator at 37°C (Thermo Fisher, USA). The lentiviral vector carrying SLC7A1-shRNA and the lentiviral control vector (GV493) were obtained from Genechem Co., Ltd. (Shanghai, China). The shRNA sequences are listed in Table S1. When the multiplicity of infection (MOI) was approximately five, SKOV3 and OVACAR3 cells were infected with the lentivirus mentioned above. The transfected cells were screened with 2.5 *μ*g/mL puromycin. Real-time fluorescence quantitative PCR and Western blotting were used to detect the knockdown efficiency of the transfected cells.

### 2.2. Real-Time Fluorescent Quantitative PCR

Total RNA was extracted from the cells using TRIzol (TaKaRa, Japan) and reverse transcribed into cDNA using a reverse transcription system (TaKaRa, Japan). A two-step real-time PCR method was applied to detect relative gene expression levels. GAPDH was used as an internal reference. The comparative threshold cycle (2^–*ΔΔ*CT^) method was used to calculate the relative gene expression level. The primer sequences used are listed in Table S2.

### 2.3. Western Blotting Assays

Total protein was obtained from cells using RIPA lysis buffer (Solarbio, Beijing, China). Proteins were isolated by electrophoresis using a 10% PAGE gel rapid preparation kit from Shanghai Yase Biomedical Technology Co., Ltd. The proteins were then transferred to PVDF membranes (Amersham, Germany) by 290 mA constant current for 120 min. After sealing with 5% skim milk powder at room temperature for 2 h, the membranes were incubated with primary anti-SLC7A1 (1 : 1000; Proteintech Cat# 14195-1-AP, RRID: AB_2190723) or anti-GAPDH (1 : 4000; Elabscience Cat# E-AB-20059, RRID: AB_2905551) antibodies. Then, each membrane was washed three times and incubated with a secondary antibody (1 : 2000; Elabscience, Wuhan, China). Protein bands were detected by enhanced chemiluminescence (Millipore, Billerica, USA).

### 2.4. Immunohistochemical Staining (IHC)

We obtained twenty ovarian cancer (serous type) tissues, five normal ovarian tissues, and five normal fallopian tubes from the Affiliated Hospital of Qingdao University. These acquisitions were approved by the Ethics Committee of the Affiliated Hospital of Qingdao University. The samples were paraffin-embedded, cut to 4-*μ*m thickness, dewaxed, and rehydrated. High-pressure antigen repair with citrate solution was performed at 120°C for 8 minutes. The cells were then washed with phosphate buffer solution (PBS) 3 times for 5 minutes each time, soaked in 3% hydrogen peroxide solution for 15 minutes, and washed with PBS 3 times for 5 minutes each time. A circle was drawn 3 mm around the tissue on each section with an immunohistochemical pen, and 10% sheep serum was sealed at room temperature for 30 minutes. Rabbit anti-SLC7A1 polyclonal antibody (1 : 50; Proteintech Cat# 14195-1-AP, RRID: AB_2190723) was then added to each sample, and samples were incubated overnight at 4°C and washed with PBS 3 times for 5 minutes each time. Secondary antibody (PV-9000) and diaminobenzidine (DAB) kits were purchased from Beijing Zhongshan Jinqiao Biotechnology Co., Ltd. The secondary antibody was added dropwise to each sample, and samples were incubated at room temperature for 30 minutes and then washed with PBS 3 times. After DAB staining at room temperature, the dye was washed with water for 20 minutes to stop the color development, and the haematoxylin was redyed and sealed. Immunohistochemical scores of SLC7A1 were performed on the tissue samples, and the staining intensity scores were 0 (negative), 1 (low), 2 (medium), and 3 (high). The staining percentage scores were 0 (no staining), 1 (1%-25% staining), 2 (26%-50% staining), and 3 (51%-100% staining). The final staining score was determined by multiplying the intensity score by the area score, ranging from 0 to 9. A final score of less than five was considered low expression or negative, and a score of 5-9 was considered medium-high expression staining.

### 2.5. Immunofluorescence (IF)

Cells were fixed on glass slides in advance on a 24-well plate. The cells on the slides were fixed with 4% paraformaldehyde (PFA) for 30 minutes and infiltrated with 0.2% Triton X-100 for 10 minutes. After they were sealed with 5% BSA at room temperature for 30 minutes, the primary antibody against CCL (1 : 100; Abcam Cat# ab45690, RRID: AB_776129) was added, and samples were incubated overnight at 4°C. Then, samples were incubated with the secondary antibody CY3 conjugated with sheep anti-rabbit IgG(H + L) antibody (1 : 2000, E-AB-1003, Elabscience Biotechnology) at room temperature for 2 hours. The slides were observed under a 400× magnification confocal laser scanning microscope (Nikon A1, Japan).

### 2.6. Colony Formation Assays

The transfected cells were seeded on a 6-well plate at a density of 1000 cells/well (NEST, China). After two weeks of culture, the cells were fixed with 4% paraformaldehyde (PFA) for 30 minutes and stained with 0.1% crystal violet (Sigma) for 30 minutes to identify colonies containing >50 cells. Counts were carried out under a light microscope. The data are expressed as the mean ± standard deviation (SD) of three independent experiments.

### 2.7. Cell Viability Assay

To detect cell proliferation, CCK-8 assays were performed based on the manufacturer's protocol (MedChem Express, China). Briefly, the cell suspensions were seeded into 96-well plates (approximately 3 × 10^3^ cells/well). Every 24 h after cell adherence, CCK-8 solution (10 *μ*L) was added to each well, and the 96-well plate was placed in an incubator at 37°C for one hour. The absorbance at 450 nm was measured with a microplate reader.

In platinum resistance assays, cell suspensions (8 × 10^3^ cells/well) were prepared and added to 96-well plates. After cell adherence, the cells were treated with various concentrations of cisplatin (MedChem Express, China) for 48 hours. CCK-8 solution (10 *μ*L) was added to each well, samples were placed in an incubator at 37°C for 2 hours, and absorbance was measured at 450 nm using a microplate reader.

### 2.8. Wound Healing Assay

The cells were plated and allowed to grow overnight to produce a fused monolayer. Two hundred-microliter tips were used to create constant-diameter scratches in each monolayer. The nonadherent cells were washed quickly with PBS. Wound images were taken at 0, 24, and 48 h using an inverted phase-contrast microscope. We used ImageJ software to measure the migration distances of cells.

### 2.9. Migration and Invasion Assays

For the invasion assays, 8.0-*μ*m transwell chambers (Corning, NY, USA) were used to determine invasion in a 24-well plate. The cells were suspended in serum-free medium (1 × 10^5^ cells/200 *μ*L) and inoculated into the upper chamber precoated with Matrigel (final concentration: 260 *μ*g/mL, BD Biosciences #356234). Then, 600 *μ*L medium containing 15% FBS was added to the lower chamber. After incubation at 37°C for 24 h, the upper chamber was removed, and the cells that had not migrated to the bottom of the upper chamber were gently wiped with a swab. Then, the transwell chamber was fixed with 4% paraformaldehyde (PFA) for 30 minutes and stained with 0.1% crystal violet for 30 minutes (Solarbio, Beijing, China). The invading cells were photographed and counted under a light microscope (Olympus) at 200× magnification. Migration assays were similar, but no Matrigel was added to the upper chamber.

### 2.10. Analysis of Intracellular Free Amino Acid Levels

At the exponential growth stage, tumor cells were selected and inoculated in ten-centimeter Petri dishes. After the cells were overgrown, 1 mL PBS was used to resuspend the cells, and a cell counting plate was used to count the cells. The concentration of the cells in the SKOV3-NC group was 1 × 10^6^ cells/mL, and that of the cells in the SKOV3-shSLC7A1 group was 5 × 10^6^ cells/mL. The amino acid concentration of the NC group was multiplied by 5 and then compared with that of the shSLC7A1 group. An appropriate amount of each sample was mixed into a 10 mL centrifuge tube, 0.02 mol/L HCl was added to dissolve the sample, and the volume was fixed. The C18 pretreatment column was activated, and the sample was purified. Twenty-microliter samples were injected into the amino acid automatic analyzer (LA8080, Hitachi High-Tech, Japan). The chromatographic column was a cationic acid resin separation column. Dual-channel detection was adopted, and the wavelengths used were 570 nm and 440 nm, respectively. The 440 nm wavelength was mainly used to detect proline. Each standard was run separately at a gradient specified by the parametric program (reaction temperature 135 ± 5°C, gradient elution analysis). With the same retention time, three consecutive processes were performed, and a *t* test was performed. The following 17 amino acids were analyzed: aspartic acid (Asp), threonine (Thr), serine (Ser), glutamic acid (Glu), glycine (Gly), alanine (Ala), cystine (Cys), valine (Val), methionine (Met), isoleucine (Ile), Leu (Leu), tyrosine (Tyr), phenylalanine (Phe), lysine (Lys), histidine (His), arginine (Arg), and proline (Pro). The absolute concentration of each amino acid is expressed in nmol/mL.

### 2.11. Expression Level Analysis

We obtained GEO transcriptome datasets (GSE14407, GSE40595) including OC and normal ovarian tissues from the Gene Expression Omnibus [17, 18] (GEO, https://www.ncbi.nlm.nih.gov/geo).

The protein expression levels of the *SLC7A1* gene in OC and normal ovarian tissues were analyzed with the Human Protein Atlas database (https://www.proteinatlas.org) [19, 20].

### 2.12. cBioPortal Database Analysis

cBioPortal (http://cbioportal.org) [21] is an open resource for visualizing and analyzing multidimensional cancer genomic data from TCGA. The database includes 1680 ovarian cancer cases from three studies (TCGA, Firehose Legacy; TCGA, Nature 2011; TCGA, PanCancer Atlas). The DNA copy number (such as “deep deletion” or “amplification”), mRNA and microRNA expression, DNA methylation, and other information about relevant genes in big data are visible.

### 2.13. Prognostic Value Analysis

The prognostic value of SLC7A1 was assessed with the Kaplan–Meier Plotter tool (http://kmplot.com) [22, 23]. The online tool calculated hazard ratios (HRs), 95% confidence intervals (CIs), and log-rank *P* values. The overall survival (OS), progression-free survival (PFS), and post-progression survival (PPS) of OC patients were obtained by classifying OC samples into high and low SLC7A1 expression groups using the optimal cut-off value. The UALCAN tool (http://ualcan.path.uab.edu/home) was used to analyze the relationship between SLC7A1 expression and OC stage and grade [24].

### 2.14. Coexpression Gene Prediction in LinkedOmics

The LinkedOmics database (http://linkedomics.org/login.php) [25] is a platform for analyzing the Cancer Genome Atlas (TCGA) data. We used the database to predict the coexpressed genes of SLC7A1 in OC. DAVID version 6.8 (https://david.ncifcrf.gov/tools.jsp) [26] is a comment, visualization, and integration found the function of the database and results in summary tools. Here, we discuss the biological processes used in gene ontology analysis [27, 28]. The DAVID tool was used for Gene Ontology (GO) and Kyoto Encyclopedia of Genes and Genomes (KEGG) pathway analyses of the top 50 positively coexpressed genes.

### 2.15. Protein-Protein Interaction (PPI)

CellPhoneDB online database (https://www.cellphonedb.org/) was used to predict ligands corresponding to SLC7A1. CellPhoneDB is an emerging database commonly used for understanding cell–cell communication, indicating the relationships between ligands and receptors based on the expression of receptors in one cell type and ligand expression in another cell type [29]. The Human Reference Protein Interactome Mapping Project (http://www.interactome-atlas.org/search) [30] is a database used to evaluate whether protein molecules interact and the strength of the interaction correlation. The two databases were used to assess the correlations of SLC7A1-CCL4 protein interactions.

### 2.16. Immune Landscape Related to SLC7A1 Expression

The correlation between SLC7A1 and immune-infiltrating cells in OC was evaluated using the EPIC algorithm in the GEPIA database (http://gepia2021.cancer-pku.cn/) [31]. The TIMER database (http://timer.cistrome.org/) [32–34] contains information on various cancer types for immune system analysis of integrated resources through various immune deconvolution methods to estimate infiltration abundance. CIBERSORT (which estimates relative abundances of RNA transcript subsets) [35] is another deconvolution algorithm that can characterize the cell composition of complex tissues from the gene expression profile. We analyzed the association between SLC7A1 and CD8^+^ T cells, CD4^+^ T cells, cancer-associated fibroblasts (CAFs), and macrophages in OC using the CIBERSORT algorithm from the TIMER database.

### 2.17. Statistical Analysis

We analyzed the data using GraphPad Prism 8.0 (GraphPad Software, Inc. Data are presented as mean ± standard deviation of at least three independent trials. Comparisons between the two groups were made using Student's *t* tests. *P* < 0.05 was considered statistically significant.

## 3. Results

### 3.1. SLC7A1 Is Overexpressed in EOC

**Figure 1:**
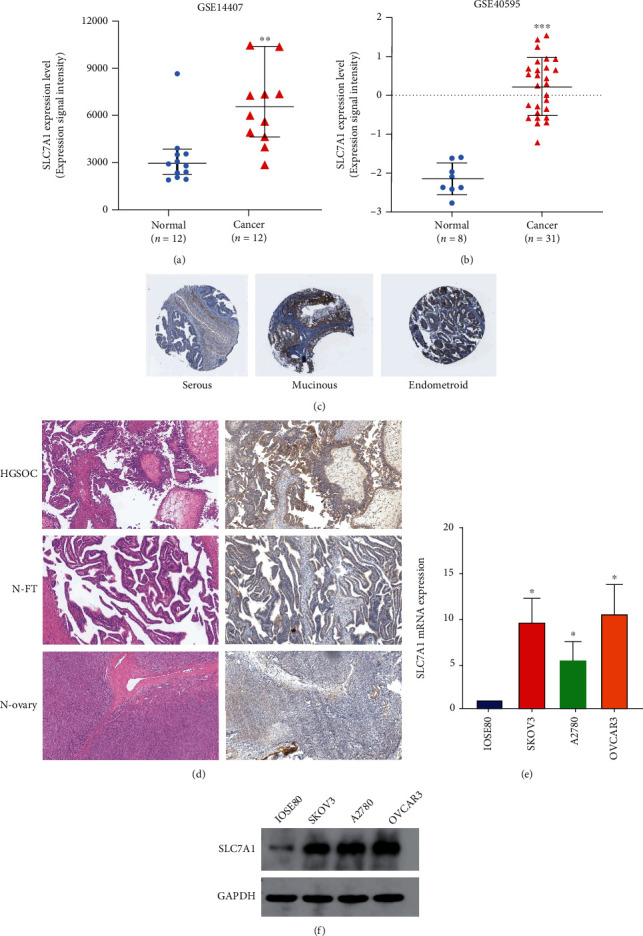
Expression levels of SLC7A1 in OC tissues and cells. (a–b) Comparison of SLC7A1 RNA expression in normal ovarian tissues (normal) and ovarian cancer tissues (cancer). The GEO database provided data for two gene expression datasets (GSE14407 and GSE40595). (c) Immunohistochemical image of SLC7A1 in OC tissue from The Human Protein Atlas database. (d) Immunohistochemistry shows a representative image of SLC7A1 protein expression in HGSOC (*n* = 20) versus that in a normal fallopian tube (N-FT) (*n* = 5) and normal ovary (N-ovary) (*n* = 5). Scale bar =100 *μ*m. (e–f) The mRNA and protein expression levels of SLC7A1 in IOSE80, SKOV3, OVCAR3, and A2780 cells were measured by qRT–PCR and Western blotting. ^∗^*P* < 0.05; ^∗∗^*P* < 0.01; ^∗∗∗^*P* < 0.001.

We searched OC-related datasets in the GEO database to study the expression of SLC7A1 in OC tissues. In the GSE40595 and GSE14407 datasets, SLC7A1 mRNA expression level was significantly higher in OC tissues than in normal ovarian tissues (Figures 1(a) and 1(b)). We identified SLC7A1 protein expression in OC tissue from the HPA database [19, 30, 36] (Figure 1(c)). SLC7A1 showed significant overexpression in serous, mucinous, and endometrioid OC tissues. Next, the expression of SLC7A1 protein in human high-grade serous ovarian cancer tissues (HGSOC) (*n* = 20), normal ovarian tissues (N-ovary) (*n* = 5), and normal fallopian tubes (N-FT) (*n* = 5) was detected by immunohistochemistry (IHC). SLC7A1 protein expression was increased in HGSOC compared with that in N-ovary and N-FT tissues (Figure 1(d)). At the cellular level, Western blotting and qRT–PCR showed that SLC7A1 expression levels at the protein and mRNA levels were higher in SKOV3, OVCAR3, and A2780 cells than in normal ovarian epithelial cells, IOSE80 (Figures 1(e) and 1(f)).

### 3.2. SLC7A1 Downregulation Affects Free Acid Uptake in EOC Cells

Current studies have shown that SLC7A1, a cationic amino acid transporter, is mainly involved in arginine transport. Nevertheless, the effect of SLC7A1 on amino acid metabolism levels in EOC is not apparent. Therefore, an automatic amino acid analyzer was used to determine whether SLC7A1 knockdown affected free amino acid uptake in SKOV3 cells. The contents (nmol) of 17 kinds of free amino acids in each milliliter of cell suspension were detected. Three independent replicates were performed. Standard curve chromatograms of 17 amino acids are shown in Figure S1. As shown in Table 1, the contents of 17 amino acids in the NC group and the shSLC7A1 group are compared. The *q* value is a Benjamini–Hochberg type statistical correction of the *P* value. The results showed that the levels of phenylalanine (Phe) and arginine (Arg) in the shSLC7A1 group were significantly decreased (*P* < 0.05). The other 15 amino acids showed no significant declines in the shSLC7A1 group (*P* > 0.05). We show differences in phenylalanine and arginine between the NC and knockdown groups in Figure S2. In conclusion, the downregulation of SLC7A1 in EOC cells inhibits the intracellular concentrations of phenylalanine and arginine, thereby affecting the amino acid metabolism of cancer cells.

### 3.3. Knockdown of SLC7A1 Inhibited the Proliferation, Invasion, and Metastasis of EOC Cells

**Figure 2:**
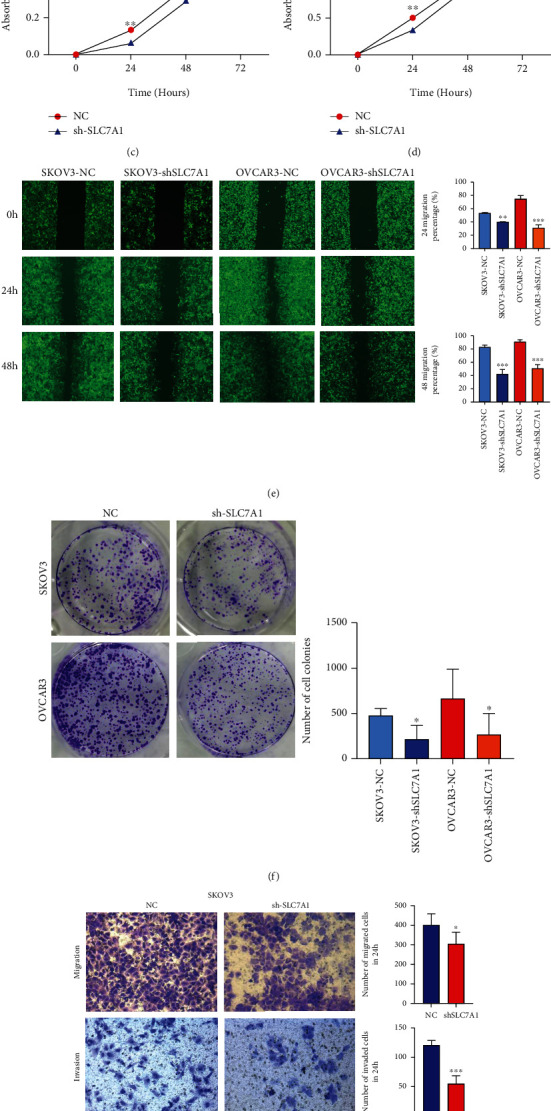
SLC7A1 regulates OC cell proliferation, invasion, and metastasis. (a–b) qRT–PCR was used to detect the knockdown efficiency of mRNA in SKOV3 and OVCAR3 cells. (c–d) CCK-8 was used to detect the proliferation of SKOV3 and OVCAR3 cells. (e) The migration of SKOV3 and OVCAR3 cells was detected by wound healing assays (*n* = 3). (f) Effects of SLC7A1 knockdown on colony formation by SKOV3 and OVCAR3 cells (*n* = 3). (g–h) Transwell assays were used to detect cell migration and invasion. Original amplification, × 200. ^∗^*P* < 0.05; ^∗^*P* < 0.01; ^∗∗∗^*P* < 0.001.

To explore whether SLC7A1 regulates the biological behavior of EOC cells, we stably transfected SKOV3 and OVCAR3 cells with SLC7A1 carried on two SLC7A1 knockdown lentiviruses (sh-SLC7A1 #1 and sh-SLC7A1 #2). The transfection efficiency was detected by quantitative RT (qRT)-PCR (Figures 2(a) and 2(b)). Finally, the sh-SLC7A1 lentivirus with the highest knockdown efficiency was selected for subsequent experiments. Western blotting assays were used to observe cell knockdown efficiency (Figure S4). The CCK-8 assays showed that SLC7A1 knockdown significantly reduced SKOV3 and OVCAR3 cell proliferation (*P* < 0.01) (Figures 2(c) and 2(d)). The wound healing experiment showed that compared with NC cells after a single scratch, scratch healing was significantly inhibited in the shSLC7A1 group (Figure 2(e)). In the cell colony formation assays, the colony formation capacity in SKOV3 and OVCAR3 cells was decreased with SLC7A1 knockdown (*P* < 0.05) (Figure 2(f)). The transwell assay indicated that the migration and invasion abilities of SKOV3 and OVCAR3 cells were significantly reduced after the SLC7A1 gene was knocked out (*P* < 0.05) (Figures 2(g) and 2(h)).

### 3.4. Knockdown of SLC7A1 Reduces Cisplatin Resistance in EOC Cells

**Figure 3:**
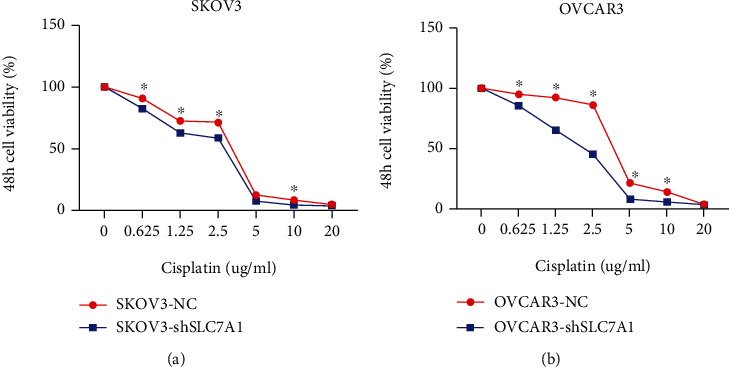
Knockdown of SLC7A1 affects the cisplatin sensitivity of ovarian cancer cells. (a–b) SLC7A1 was knocked down in SKOV3 and OVCAR3 cells and cultured in different concentrations of cisplatin for 48 h. ^∗^*P* < 0.05; ^∗^*P* < 0.01; ^∗∗∗^*P* < 0.001.

Cisplatin resistance is one of the causes of EOC recurrence [37]. Therefore, we investigated whether SLC7A1 is related to the response of EOC to cisplatin. Knockdown of SLC7A1 increased the cisplatin sensitivity of EOC cells (*P* < 0.05) (Figures 3(a) and 3(b)). Figure S3 illustrates the sensitivity of SKOV3 and OVCAR3 cells to cisplatin at different concentrations and rates of concentration change.

### 3.5. SLC7A1 Was Coexpressed with Other Genes in OC

**Figure 4:**
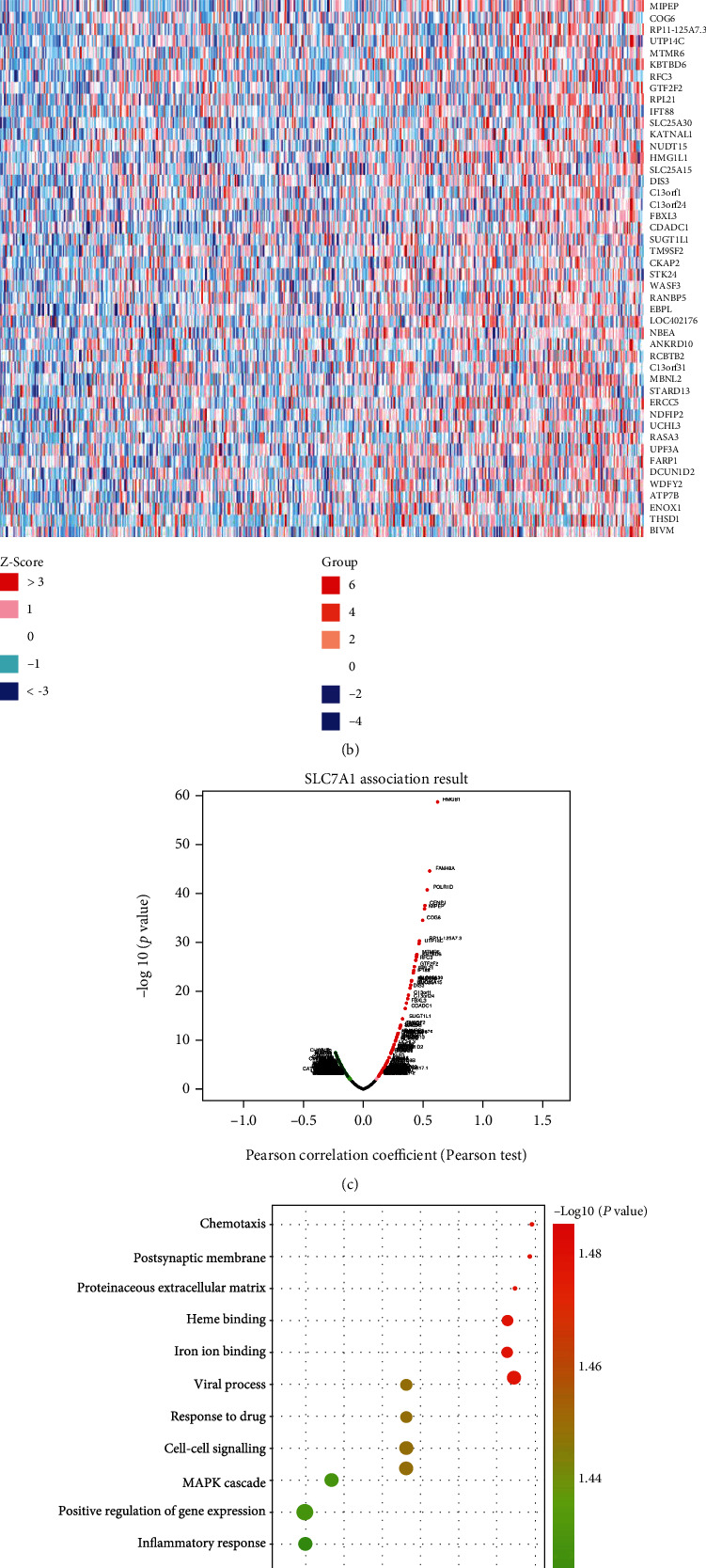
Coexpression network of SLC7A1 in OC. (a) The heat map shows the top 50 genes negatively associated with SLC7A1 expression in the OC dataset (TCGA database). (b, c) The heat map and volcano map show the top 50 genes positively coexpressed with SLC7A1. (d) The bubble diagram shows the GO analysis of the top 50 coexpressed genes with positive correlations. -log_10_(*P* value) >1.4. (e) The KEGG analysis of the top 50 coexpressed genes with positive correlations is shown in the bubble map, and those with -log_10_(*P* value) >1.3 were selected. GO: gene ontology; KEGG: Kyoto Encyclopedia of Genes and Genomes.

We used the functional module of LinkedOmics to construct the coexpression network of SLC7A1 to clarify the biological function of SLC7A1.  Figure 4(a) represents the top 50 genes negatively related to SLC7A1 expression in OC, among which SLC7A1 had a strong negative correlation with the ubiquitin ligase CHAF1B. Figures 4(b) and 4(c) illustrate the top 50 genes positively associated with SLC7A1 expression in OC with heat and volcano maps. Notably, SLC7A1 showed a strong positive correlation with the oncogenic protein HMGB1. GO term annotations were performed for the top 50 positively related genes (Figure 4(d)). The results showed that SLC7A1-coexpressed genes were mainly involved in protein heterodimerization activity, the inflammatory response, the MAPK signalling pathway, and cell–cell signal transduction (*P* < 0.05). KEGG pathway analysis showed that SLC7A1-coexpressed genes were primarily involved in cancer pathways, MAPK signalling pathways, calcium ion dysregulation, and transcription dysregulation in cancer (*P* < 0.05) (Figure 4(e)).

### 3.6. Prognostic Value of SLC7A1 in OC

**Figure 5:**
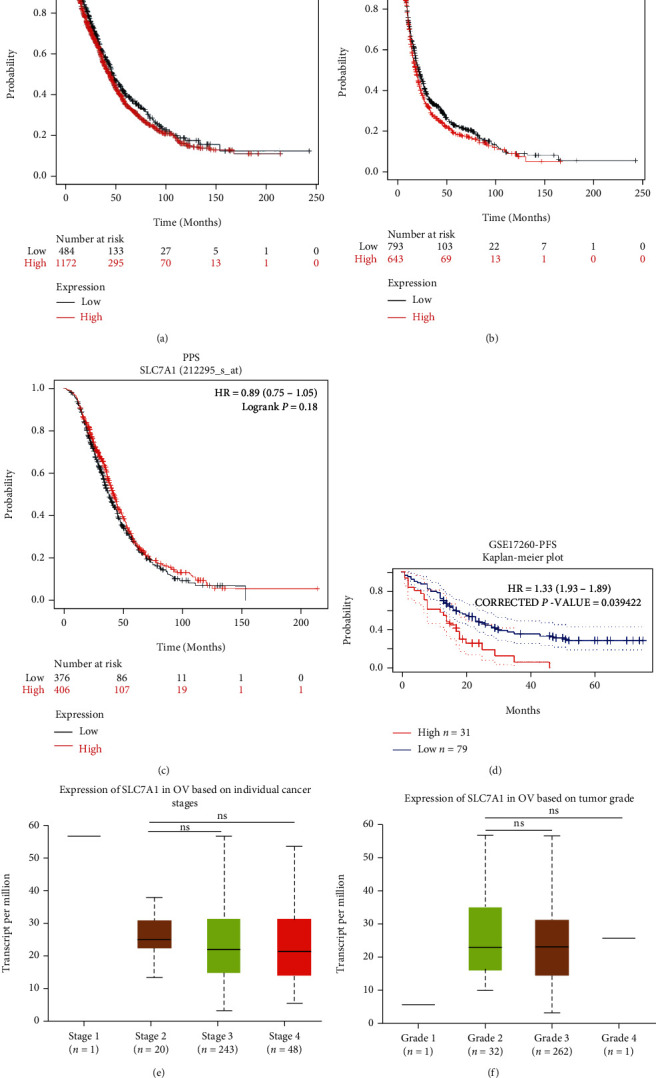
The prognostic value of SLC7A1 in OC patients. (a–c) Correlations of SLC7A1 expression level with OS, PFS, and PPS in OC patients in the GSE212295 dataset. (d) Kaplan–Meier survival curves of high and low SLC7A1 expression in the GSE17260 dataset were compared. (e–f) The relationships between SLC7A1 mRNA expression level and tumor stage and grade in TCGA samples. ^∗^*P* < 0.05; ^∗∗^*P* < 0.01; ^∗∗∗^*P* < 0.001; ns: not significant; HR: hazard ratio; OS: overall survival; PFS: progression-free survival; PPS: post-progression survival; OC: ovarian cancer.

**Figure 6:**
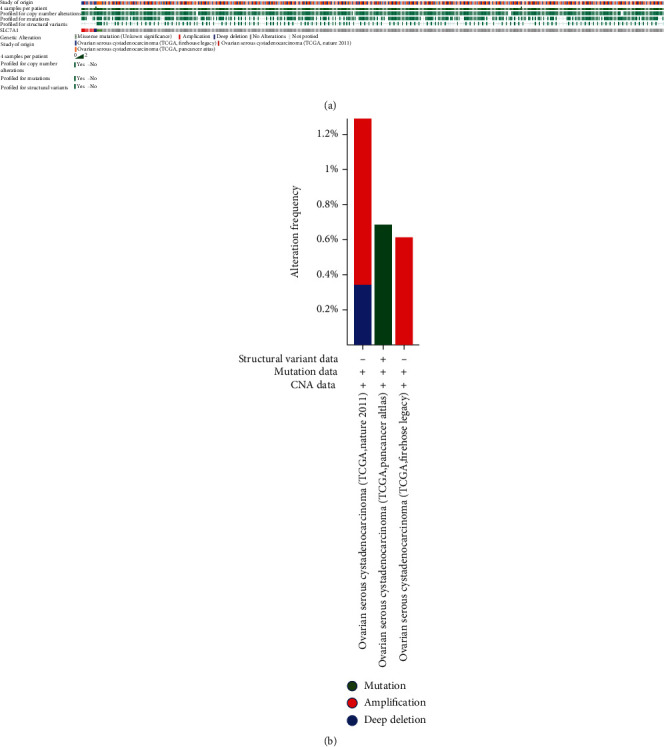
Analysis of SLC7A1 gene variation in patients with ovarian cancer (cBioPortal). (a) OncoPrint summarizes the genetic variation in SLC7A1 in serous ovarian cancer. Three types of genetic alterations were defined: missense mutation, amplification, and deep deletion. (b) The frequencies of modification, amplification, and deep deletion of SLC7A1 in serous ovarian cancer are summarized. CAN: copy number alteration; TCGA: the Cancer Genome Atlas.

In the GSE212295 dataset, the Kaplan–Meier survival curve showed that SLC7A1 overexpression was associated with adverse PFS in OC patients (*P* = 0.039). There was no significant correlation with OS or PPS (*P* > 0.05) (Figures 5(a)–5(c)). Meanwhile, in the GSE17260 dataset, compared with OC patients with lower expression levels, OC patients with higher SLC7A1 expression levels also experienced poorer PFS (*P* < 0.05) (Figure 5(d)). We found no significant correlations between SLC7A1 mRNA expression and the stage or grade of OC patients in TCGA (*P* > 0.05) (Figures 6(e) and 6(f)).

### 3.7. Genetic Variants of SLC7A1 Are Involved in OC Progression

To determine whether the upregulation of SLC7A1 in OC tissue is caused by genetic variation, we used the cBioPortal tool to measure the frequency of SLC7A1 changes in DNA sequence data in OC. The database contained 1,680 serous ovarian cancer cases from three studies (TCGA, Nature 2011; TCGA, PanCancer Atlas; TCGA, Firehose Legacy). In this study, the sequence variation of SLC7A1 in ovarian cancer accounted for 0.9% of the total sample (Figure 6(a)). The rates of change in SLC7A1 in samples from TCGA (Nature 2011, PanCancer Atlas, and Firehose Legacy) were 0.6%, 0.7%, and 1.4%, respectively (Figure 6(b)). Amplification accounted for most genetic variation (0.6%, TCGA, Nature 2011; 1.08%, TCGA, Firehose Legacy). Therefore, amplification may be one of the main mechanisms of SLC7A1 overexpression in ovarian cancer.

### 3.8. SLC7A1 Was Associated with the Tumor Immune Microenvironment in OC Patients

**Figure 7:**
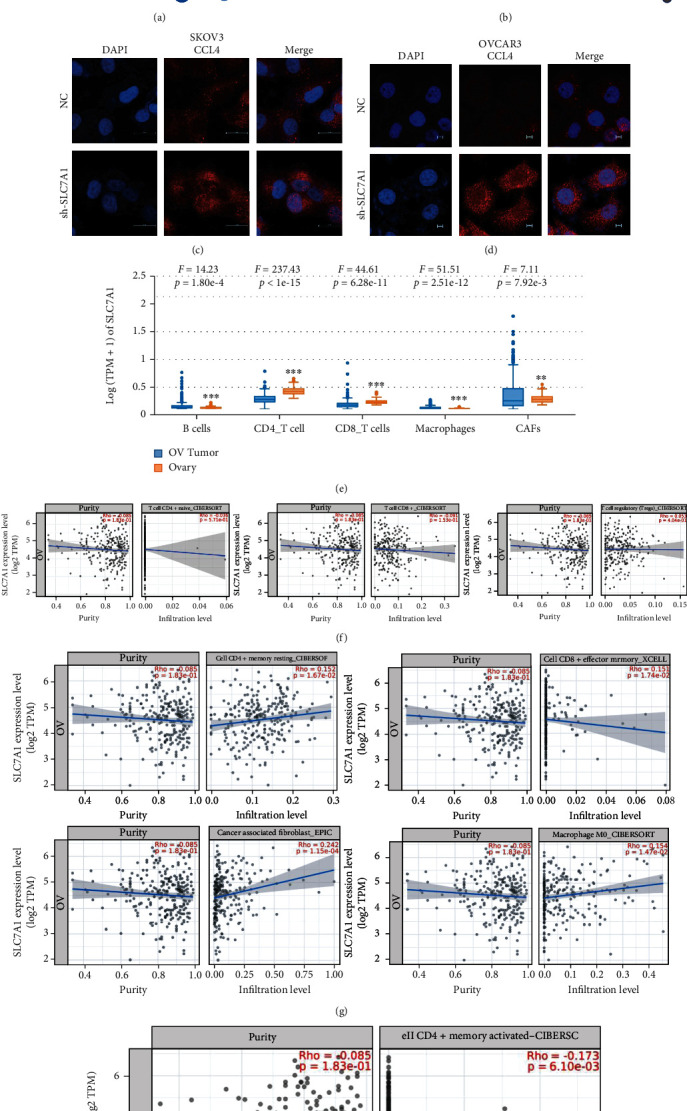
Relationship between SLC7A1 and the immune microenvironment in OC. (a–b) The Human Reference Protein Interactome Mapping Project database predicted the correlation between SLC7A1 and CCL4. (c–d) The expression level of CCL4 after SLC7A1 knockdown was detected by immunofluorescence in SKOV3 and OVCAR3 cells. The scales are 20 *μ*m and 5 *μ*m, respectively. (e) The EPIC method was used to calculate the correlations of the interstitial and immune scores of TCGA-OV patients with SLC7A1 expression. (f–h) Scatterplots showing the correlations of the ratios of eight kinds of TICs with SLC7A1 expression. The blue lines indicate the relationships between immune cell proportions and SLC7A1 expression. Pearson coefficients were used for correlation tests. ^∗^*P* < 0.05; ^∗∗^*P* < 0.01; ^∗∗∗^*P* < 0.001; TCGA-OV: the Cancer Genome Atlas-Ovarian Cancer.

Studies have shown that the expression of SLC7A1 on T cell membranes is involved in the differentiation and development of T cells. On the other hand, the CellPhoneDB database and the Human Reference Protein Interactome Mapping Project database suggested that SLC7A1 and CCL4 interact through a ligand–receptor binding relationship. A predicted correlation coefficient of 0.7 indicated a strong correlation between SLC7A1 and CCL4 in OC (Figures 7(a) and 7(b)). Furthermore, cell immunofluorescence showed that the expression of CCL4 was significantly increased in SKOV3-shSLC7A1 and OVCAR3-shSLC7A1 cells compared with the NC group, especially around the cell membrane (Figures 7(c) and 7(d)). Considering that CCL4 acts as a chemokine, we hypothesized that SLC7A1 is involved in regulating tumor-infiltrating immune cells (TICs). We found that SLC7A1 was correlated with immune components in the tumor microenvironment and highly enriched in cancer-associated fibroblasts (CAFs) (*P* = 0.00792) (Figure 7(e)). The CIBERSORT algorithm evaluated the correlations of SLC7A1 with the expression of TICs in TCGA OC samples in the TIMER database. As shown in Figure 7(f), there are no apparent correlations with CD4^+^ naive T cells, CD8^+^ T cells, or T regulatory cells (Tregs) (*P* > 0.05). However, as observed in Figure 7(g), there were significant positive correlations with CD4^+^ resting memory cells, CD8^+^ effector memory T cells, cancer-associated fibroblasts (CAFs), and M0 macrophages (*P* < 0.05). As shown in Figure 7(h), SLC7A1 was negatively correlated with CD4^+^ memory-activated cells (*P* = 0.0061).

## 4. Discussion

As the second-largest membrane protein family and the largest transporter family, the solute carrier (SLC) superfamily is a group of proteins that can transport various substances, including ions, drugs, and metabolites, via electrochemical or ion gradient-mediated transmembrane transport [38, 39]. SLC7A1, also known as CAT-1, is highly expressed in some tumor tissues and cells, promoting the occurrence and development of tumors. SLC7A1 gene expression in the human breast cancer cell lines McF-7 and T47D has been found to be higher than that in normal breast cell lines, and apoptosis of McF-7 and T47D cells was significantly increased after SLC7A1 gene knockdown [14]. SLC7A1 mRNA and protein levels have been found to be higher in colorectal cancer (CRC) tissues than in normal tissues, and the application of an anti-SLC7A1 monoclonal antibody in colorectal cancer cell lines showed prominent antitumor activity [10]. Our study comprehensively explored the association between the biological function of SLC7A1 and drug resistance in OC based on bioinformatics analysis and in vitro experiments. The results showed that SLC7A1 overexpression in EOC cells promoted migration, invasion, metastasis, and resistance to cisplatin. In addition, the survival curve suggested that the high expression of SLC7A1 in OC was related to the adverse PFS of patients (*P* < 0.05). Therefore, SLC7A1 is a promising target for ovarian cancer treatment.

SLC7A1 has been widely studied in hepatocellular carcinoma (HCC). Some studies have shown that miRNA-122 promotes the sensitivity of HCC to sorafenib by targeting downstream SLC7A1 [40]. Arginine uptake is solely dependent on SLC7A1, and targeting SLC7A1 or arginine restriction could be novel therapeutic strategies for treating HCC [41]. In EOC, arginine deprivation induces SKOV3 cell death through autophagy, reducing cell migration and adhesion [42]. Our study found that knockdown of SLC7A1 inhibited the contents of arginine and phenylalanine in SKOV3 cells and increased the sensitivity of EOC cells to cisplatin. We hypothesize that the decrease in phenylalanine may be due to the remodelling of cellular energy metabolism secondary to the decrease in arginine content. Therefore, SLC7A1 is involved in the remodelling of amino acid metabolism in EOC. Targeting SLC7A1 can be used as a new strategy in cisplatin therapy, and the expression level and function of miRNA-122 in EOC cells may need to be further explored.

On the other hand, SLC7A1 is not only highly expressed in some tumors and involved in tumor progression but also participates in the activation and proliferation of T cells through transmembrane transport of arginine, which is a new mechanism for regulating tumor immune escape and adaptive immune responses [43]. SLC7A1 mRNA levels were specifically upregulated in naive and memory CD4^+^ T cells and CD8^+^ T cells. Arginine uptake and proliferation of T cells have been found to be impaired when SLC7A1 expression was inhibited [16]. Furthermore, Huang et al. [44] used CRISPR technology to reveal the important nutritional signalling pathway involved in the differentiation and development of CD8^+^ T cells. SLC7A1 and SLC38A2 inhibited the differentiation of CD8^+^ memory T cells (TMEM) by partially upregulating mTORC1 activity [44]. As a protein highly expressed in OC, we found that SLC7A1 was positively correlated with CD4^+^ resting memory cells, CD8^+^ effector memory cells, cancer-associated fibroblasts (CAFs), and M0 macrophages in ovarian cancer by immune evaluation (*P* < 0.05). SLC7A1 was negatively correlated with CD4^+^ activated memory cells (*P* < 0.05), suggesting that SLC7A1 expression was related to the activation of CD4^+^ T cells in the immune microenvironment and that SLC7A1 could be used as a potential marker for immunotherapy in OC. We noted a significant correlation between SLC7A1 and CAFs in the TIMER database (*P* = 0.00015), and immunohistochemical results also suggested that SLC7A1 was highly expressed not only in tumor tissues but also in interstitial tissues in EOC. Considering that the high expression of CAFs is considered to promote cancer and participates in various biological processes in the tumor microenvironment (TME) [45], the role of SLC7A1 in CAFs is worthy of further study.

As a chemokine, CCL4 is essential for the immune response in the tumor microenvironment. In melanoma, extensive RNA sequencing data has shown that SLC7A1-CCL4 was significantly downregulated in activated CD8^+^ T cell/DC/proliferating T cell/tumor cell interactions in treated nonresponders during PD-1 inhibitor therapy [46]. In our study, SLC7A1 overexpression inhibited the expression of the chemokine CCL4, suggesting that downregulation of CCL4 due to SLC7A1 overexpression may be one of the causes of EOC immunotherapy tolerance. Previous studies have shown significantly lower serum levels of CCL4/MIP-1*β* in patients with EOC compared to healthy women (*P* = 0.03) and associated with CA125 levels [47]. This is consistent with the downregulation of CCL4 by SLC7A1 overexpression and poor PFS in EOC patients. However, specifically how CCL4 plays a role in the immune escape of EOC remains unclear.

## 5. Conclusions

Our study showed that SLC7A1 was highly expressed in EOC cells, promoting the proliferation, invasion, migration, and cisplatin resistance of cells, and was associated with poor prognosis. SLC7A1 not only participates in the metabolic remodelling of free amino acids in EOC cells but also inhibits the expression of the CCL4 molecule, which is closely related to the immune infiltration microenvironment of the tumor. Therefore, SLC7A1 is expected to be a potential biomarker for targeted EOC therapy. However, this study lacked in vitro experiments, and the significance of the high expression of SLC7A1 in CAFs and the specific role of CCL4 in immune escape are not clear, so further experimental exploration is needed.

## Figures and Tables

**Table 1:** Concentration of free amino acids in SKOV3-NC and SKOV3-shSLC7A1 groups.

Amino acid (nmol/mL)	SKOV3-NC	SKOV3-shSLC7A1	*P* value	*q* value
Aspartate	3.65 ± 17.47	2.187 ± 2.64	0.4461	0.5417
Threonine	25.37 ± 99.4	12.02 ± 61.9	0.2846	0.4220
Serine	4.17 ± 5.40	3.13 ± 7.30	0.2797	0.4220
Glutamate	25.42 ± 64.80	24.40 ± 40.02	0.9458	0.9458
Glycine	30.98 ± 124.83	36.90 ± 228.70	0.7988	0.8487
Alanine	27.43 ± 75.91	31.35 ± 138.49	0.7821	0.8487
Cystine	0.625 ± 2.22	0.35 ± 1.91	0.3552	0.4645
Valine	3.60 ± 7.62	1.35 ± 0.64	0.0647	0.2200
Methionine	1.70 ± 0.64	0.98 ± 0.32	0.2856	0.4220
Isoleucine	2.28 ± 4.13	1.00 ± 0.64	0.0605	0.2200
Leucine	2.70 ± 3.81	1.65 ± 1.27	0.0800	0.2267
Tyrosine	1.35 ± 5.72	0.65 ± 0.64	0.2621	0.4220
Phenylalanine	0.43 ± 0.32	0.15 ± 0.00	0.0082	0.0366<0.05
Lysine	4.10 ± 6.35	1.90 ± 0.64	0.0484	0.2200
Histidine	2.95 ± 10.8	0.93 ± 0.32	0.1402	0.3405
Arginine	18.03 ± 13.02	5.28 ± 0.317	0.0064	0.0366<0.05
Proline	4.95 ± 5.72	2.63 ± 9.21	0.2979	0.4220

## Data Availability

Some of the data supporting my findings can be found at the hyperlink to the manuscript. The data generated during the experiment are available on reasonable request from the corresponding author.

## Supplementary Materials

Supplementary Materials Figure S1: The chromatograms of seventeen kinds of free amino acids in standard substances were detected for two-channel detection by the amino acid automatic analyzer. Figure S2: Differences in phenylalanine (Phe) and arginine (Arg) between the NC and shSLC7A1 groups in SKOV3 cells. Figure S3: Effects of cisplatin concentration and rate of change in concentration on responses of SKOV3 and OVCAR3 cells to cisplatin. Figure S4: Western blotting was used to detect SLC7A1 knockdown efficiency in SKOV3 and OVCAR3 cells. Table S1: Sequence of SLC7A1 shRNA. Table S2: Primers used in real-time PCR, including the primer sequences for GAPDH and SLC7A1. ^∗^*P* < 0.05; ^∗∗^*P* < 0.01; ^∗∗∗^*P* < 0.001.
