# Supplementary material for: SLC7A1 Overexpression Is Involved in Energy Metabolism Reprogramming to Induce Tumor Progression in Epithelial Ovarian Cancer and Is Associated with Immune-Infiltrating Cells
Source: J Oncol. 2022 Sep 12;2022:5864826. doi: 10.1155/2022/5864826 (PMC9484923; doi:10.1155/2022/5864826)
Supplement: Supplementary Materials — Figure S1: The chromatograms of seventeen kinds of free amino acids in standard substances were detected for two-channel detection by the amino acid automatic analyzer. Figure S2: Differences in phenylalanine (Phe) and arginine (Arg) between the NC and shSLC7A1 groups in SKOV3 cells. Figure S3: Effects of cisplatin concentration and rate of change in concentration on responses of SKOV3 and OVCAR3 cells to cisplatin. Figure S4: Western blotting was used to detect SLC7A1 knockdown efficiency in SKOV3 and OVCAR3 cells. Table S1: Sequence of SLC7A1 shRNA. Table S2: Primers used in real-time PCR, including the primer sequences for GAPDH and SLC7A1. ∗P < 0.05; ∗∗P < 0.01; ∗∗∗P < 0.001. [file 5864826.f1.docx]

**SUPPLEMENTARY FIGURES AND TABLES**


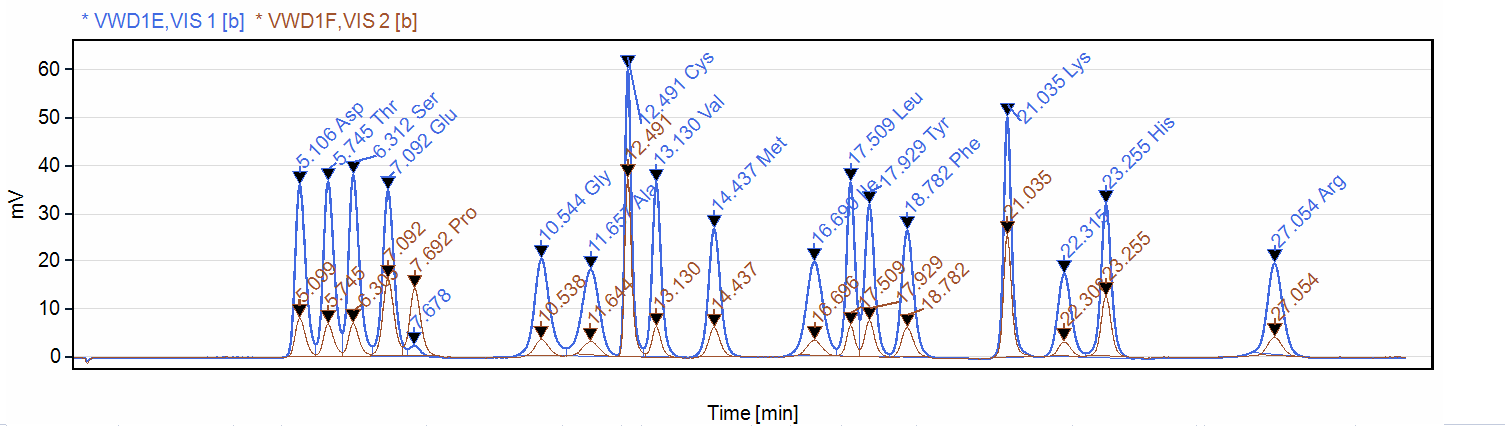


**Supplementary Figure S1.**Standard curve chromatograms of 17 amino acids are shown.The chromatograms of 17 kinds of free amino acids in standard substances were detected for two-channel detection by the amino acid automatic analyzer**.**The blue line (VWD1E, VIS 1[b]) represents the wavelength at 570nm, and the yellow line (VWD1F, VIS 2[b]) represents the wavelength at 440nm, in which the 440nm wavelength mainly detects the content of free proline. Retention times of 5.11, 5.75, 6.31, 7.09, 7.69, 10.54, 11.66, 12.49, 13.13, 14.44, 16.69, 17.51, 17.93, 18.78, 21.04, 23.25 and 27.05min correspond to aspartate (Asp), threonine (Thr), serine (Ser), Glutamic acid（Glu）, proline (Pro)，glycine (Gly), alanine (Ala), cysteine (Cys), valine (Val), methionine (Met), isoleucine (Ile), leucine (Leu), tyrosine (Tyr), phenylalanine (Phe), lysine (Lys), histidine (His) and arginine (Arg) respectively.

**Supplementary Figure S2.**The concentrations of phenylalanine(Phe) and arginine(Arg) in SKOV3 cells were compared between NC and shSLC7A1 groups. *p<0.05;**p<0.01;***p<0.001.

**Supplementary Figure S3.** Effects of cisplatin concentration and rate of change in concentration on responses of SKOV3 and OVCAR3 cells to cisplatin.*p<0.05;**p<0.01;***p<0.001.


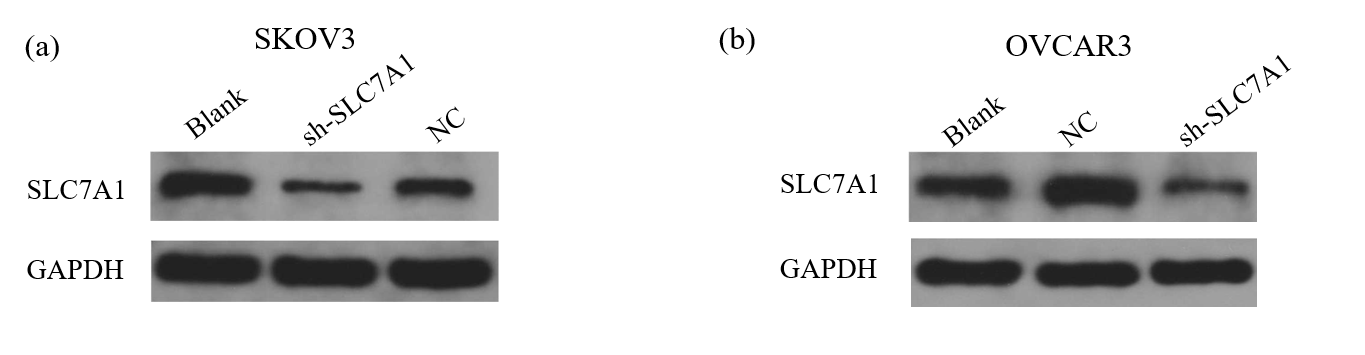


**Supplementary Figure S4.** Western blotting experiments were used to detect SLC7A1 knockdown efficiency in SKOV3 and OVCAR3 cells.

**Supplementary table S1.** Sequences of shRNA

| **shRNA Name** | **Sense** | **Antisense** |
| --- | --- | --- |
| SLC7A1#1 | CCGGCTGGGCTAATTGTGAACATTTCTCGAGAAATGTTCACAATTAGCCCAGTTTTTG | AATTCAAAAACTGGGCTAATTGTGAACATTTCTCGAGAAATGTTCACAATTAGCCCAG |
| SLC7A1#2 | CCGGGCTGAGGATGGACTGCTATTTCTCGAGAAATAGCAGTCCATCCTCAGCTTTTTG | AATTCAAAAAGCTGAGGATGGACTGCTATTTCTCGAGAAATAGCAGTCCATCCTCAGC |

**Supplementary table S2.** Primers used in real-time PCR.

| **Gene name** | **Forward (5'-3')** | **Reverse (5'-3')** |
| --- | --- | --- |
| GAPDH | GGAGCGAGATCCCTCCAAAAT | GGCTGTTGTCATACTTCTCATGG |
| SLC7A1 | CACCAACTGGGACGACAT | AGGCGTACAGGGATAGCA |
